# Evaluating the shift in psychiatric care: Associations between remote consultation use and clinical outcomes in a large longitudinal cohort

**DOI:** 10.1038/s41746-026-02922-w

**Published:** 2026-06-26

**Authors:** Liliana Hidalgo-Padilla, Robert Stewart, Matthew Broadbent, Mariana Pinto da Costa

**Affiliations:** 1 Institute of Psychiatry, Psychology & Neuroscience, King’s College London, London, UK; 2 South London and Maudsley NHS Foundation Trust, London, UK

**Keywords:** Diseases, Health care, Medical research, Psychology, Psychology

## Abstract

The shift to remote consultations represents a profound change in mental health service delivery, comparable to the historic closure of long-stay institutions, yet it occurred without formal evaluation. We aimed to identify diagnostic groups for whom remote care is safe and those for whom it may carry risk. We conducted a retrospective study assembling five annual cohorts using electronic health records from South London, including adults with psychiatric diagnoses (ICD-10 “F” codes) receiving community/outpatient care between March 2019 and March 2024. We examined associations between 10% increments in the proportion of remote consultation use and five outcomes: psychiatric hospitalisation, psychiatric assessments in emergency care, home treatment team contacts, and clinical mentions of improvement or deterioration. Logistic regression models were adjusted for demographic, social, and clinical factors, and stratified by diagnosis. Based on 106,153 observations across five cohort-years, higher use of remote consultations was not associated with worse outcomes for most psychiatric diagnoses, suggesting that remote care is non-inferior to in-person care. However, greater remote care use in patients with schizophrenia-related disorders (F20-F29) was associated with increased odds of hospitalisation (OR: 1.06 [1.03–1.09]), and emergency assessment (OR: 1.04 [1.01–1.07]). While remote mental health consultations are generally safe, but clinical caution is warranted for schizophrenia-related disorders.

## Introduction

The shift to remote care is possibly the most profound change in mental health service delivery since the deinstitutionalisation process, where long-stay psychiatric institutions started being closed systematically to give space to a community-based approach. By using various technologies, such as video calls, phone calls, or text messaging, remote mental health consultations enabled specialists to assess, treat, and follow up patients^1^ and maintain clinical service provision during the COVID-19 health crisis. However, unlike deinstitutionalisation, which took several decades to implement^2^, the digital shift happened quite rapidly due to necessity^3–6^, rather than being driven by clinical evidence. However, despite being widely integrated into health systems worldwide, remote consultations have not been formally evaluated at scale in a post-pandemic world.

Remote consultations have shown to be cost-effective and satisfying for patients^7,8^, have helped reduce stigma, promote patient empowerment, enhance treatment adherence^9^, and have increased access to care for individuals living in rural areas^4,5^. Early evidence and controlled studies have reported similar efficacy levels of remote and face-to-face mental health consultations in healthcare settings^3–6,9–11^, especially for patients with depression and serious mental illness, such as schizophrenia and bipolar disorder, who have shown improved outcomes for remote consultations compared to face-to-face^12–16^.

However, these findings cannot predict the safety of remote care as a routine delivery model in the current context because they derive from small sample sizes or highly controlled settings, which makes generalisability problematic; or were conducted before or during the acute phase of the pandemic, which may reflect contexts where remote care was either offered to stable patients only or enforced due to a temporary crisis. There is growing concern that as remote consultations become part of the standard of care, the specific needs of patients with understudied diagnoses or with more severe symptoms or conditions may be overlooked, especially when remote consultations pose some challenges including the difficulty in assessing non-verbal cues or weakened therapeutic alliance^5,7,9^.

While randomised controlled trials allow to establish causality, they are challenging in a context where remote technologies are ubiquitous and embedded in standard care. Therefore, there is an urgent need to conduct updated, large-scale observational evidence that moves beyond the critical pandemic phase to evaluate the long-term safety of remote consultations across the full spectrum of psychiatric diagnoses.

To address this knowledge gap, we conducted an exploratory large-scale evaluation of remote consultations using electronic health records across psychiatric diagnoses to investigate the association between remote consultation use and clinical outcomes and identify diagnostic groups for whom remote care is safe and those for whom it may carry risk. Despite this being an exploratory study, based on the literature described above, we anticipated that patients with depression and serious mental illness would show improved outcomes.

## Results

### Descriptive statistics

**Fig. 1:**
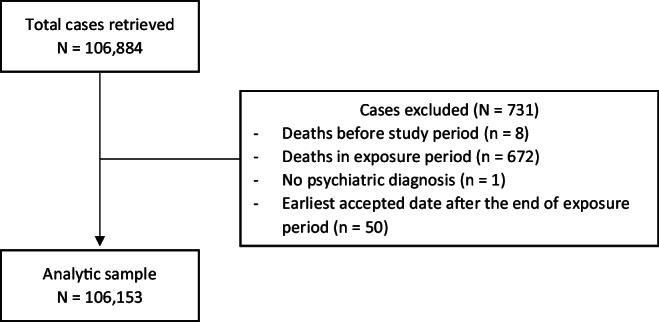
Flow diagram for study population selection. Flowchart of cases retrieved, cases excluded due to exclusion criteria, and final analytic sample number.

We retrieved 106,884 records from the Clinical Interactive Record System (CRIS) from the South London and Maudsley NHS Foundation Trust (SLaM). After applying exclusion criteria, the combined cohorts included 106,875 person-year observations, where each observation had an ICD-10 “F” diagnosis and at least one consultation during the first quarter (Q1) of a study year (see Fig. 1), and with an average of 269 follow-up days.

The cohort characteristics for each year, from March 2019 to March 2024, are detailed in Table 1. The 2020 cohort was distinct, characterised by a smaller sample size reflecting the decrease in the total consultations at the onset of the COVID-19 pandemic. Compared to other years, the 2020 cohort included lower proportions of female and older patients, but a higher proportion of those with recorded loneliness. The same cohort showed higher proportions of patients with substance use (F10-F19) and schizophrenia-related disorders (F20-F29), while the number of patients diagnosed with dementia, depression, phobias, post-traumatic stress disorder, and eating disorders was lower. Finally, patients in that cohort also showed increased clinical severity, with higher proportions of psychiatric hospitalisations, emergency assessments, home treatment team contacts, and psychotropic medication use.

**Table 1:** Sample characteristics by year

	Cohort year				
	2019	2020	2021	2022	2023
*N*	22,082	14,779	23,517	22,707	23,068
**Age group (%)**^1^					
18–25	14.0	14.3	15.6	14.7	14.3
26–34	19.0	19.1	19.8	19.4	19.0
35–49	29.1	30.1	27.8	27.5	27.1
50–69	26.7	27.7	26.4	27.2	27.3
70+	11.2	8.8	10.5	11.2	12.2
**Sex (%) (*N*** = **106,598)**^**1**^					
Female	50.2	47.5	51.9	52.1	52.3
Male	49.8	52.5	48.1	47.9	47.7
**Ethnic group (%)**^**1**^					
White	50.4	49.2	48.1	48.8	48.6
Black	24.8	27.7	26.0	26.8	27.1
Asian	5.6	5.5	5.6	5.7	6.1
Mixed	3.5	3.5	4.1	4.4	5.2
Other ethnic group	4.6	4.4	5.2	5.1	5.4
Not stated	11.1	9.6	11.0	9.2	7.6
**IMD quintiles (%) (*n*** = **104,000)**^**1**^					
1 - more deprived	19.5	20.3	20.0	20.0	20.6
2	20.4	19.8	20.2	20.0	19.8
3	19.5	20.2	19.9	20.1	20.0
4	20.6	20.0	20.0	19.8	19.9
5 - less deprived	20.1	19.7	20.0	20.0	19.8
**Marital status (%)**^**1**^					
No partner	65.5	65.9	61.2	59.7	56.8
Partner	13.6	11.8	11.6	11.7	11.7
Not stated	20.9	22.3	27.2	28.6	31.5
**Loneliness (%)**^**2**^					
Not recorded	85.8	81.3	85.4	84.8	84.4
Recorded	14.2	18.7	14.6	15.2	15.6
**Long-term condition (%)**^**2**^					
No	26.6	24.3	25.3	25.6	38.3
Yes	73.4	75.7	74.7	74.4	61.7
**Mobility impairment (%)**^**2**^					
No	98.1	98.0	98.2	98.3	98.5
Yes	1.9	2.0	1.8	1.7	1.5
**Hearing impairment (%)**^**2**^					
No	99.5	99.5	99.5	99.6	99.6
Yes	0.5	0.5	0.5	0.4	0.4
**Visual impairment (%)**^**2**^					
No	99.6	99.6	99.7	99.7	99.6
Yes	0.4	0.4	0.3	0.3	0.4
**Diagnosis (%)**^**1**^					
F00-F03: Dementia	5.7	3.7	5.4	5.6	6.5
F04-F09: Other disorders due to substances, brain damage or physical disease	2.7	2.4	2.5	2.8	3.2
F10-F19: Mental and behavioural disorders due to psychoactive substance use	14.0	16.4	13.2	13.6	13.4
F20-F29: Schizophrenia, schizotypal and delusional disorders	25.1	31.1	24.8	25.5	24.6
F30-F31: Manic episode and bipolar affective disorder	4.8	5.2	5.0	5.0	4.8
F32-F39: Depressive episode, recurrent depressive disorder and other mood affective disorders	10.1	7.4	8.9	8.3	7.4
F40-F41: Phobic and other anxiety disorders	4.5	3.4	4.4	4.4	4.2
F43: Reaction to severe stress and adjustment disorders	3.7	3.0	3.7	3.6	3.7
F50: Eating disorders	2.1	1.8	2.2	2.6	2.8
F60-F61: Specific, mixed and other personality disorders	5.7	5.4	5.8	5.5	5.3
Other	10.5	9.2	10.0	9.9	10.5
Unspecified	11.2	10.8	14.0	13.1	13.7
**Referral recency (%)**^1, *^					
Recent	35.5	32.6	33.9	33.0	31.6
Intermediate	34.8	33.1	33.0	32.5	33.3
Long-standing	29.8	34.4	33.1	34.5	35.1
**Severity - hospitalisations (%)**^**1**^					
No	92.2	90.1	92.2	91.7	92.5
Yes	7.8	9.9	7.8	8.3	7.5
**Severity – emergency assessment (%)**^**1**^					
No	87.1	84.2	86.5	85.4	85.7
Yes	12.9	15.8	13.5	14.6	14.3
**Severity – home treatment team (%)**^**1**^					
No	92.9	90.8	92.1	92.2	92.8
Yes	7.1	9.2	7.9	7.8	7.2
**Severity - medication (%)**^**2**^					
None	37.6	32.0	33.5	34.9	35.1
1	31.6	33.7	33.5	33.0	33.0
2+	30.8	34.3	33.1	32.0	32.0

^1^Data derived from structured EHR fields.

^2^NLP-derived data.

*Recency derived from tertiles.

### Use of remote consultations

The use of remote consultations peaked in 2020 and 2021 and settled in the following years, as detailed in Table 2. However, uptake was not equitable across demographic groups (see Supplementary Table 1). Remote care was significantly more common among younger and female patients. In contrast, Black individuals, patients without partners, and those with recorded loneliness had significantly lower odds of high remote uptake. Regarding clinical factors, patients with long-term conditions, mobility impairments and those with schizophrenia-related (F20–F29) and substance use disorders (F10–F19) had lower proportions of remote consultation use, whereas usage was higher among patients with eating disorders. Patients with long-standing referral and those with markers of higher severity, including frequent baseline psychiatric hospitalisations, emergency assessments, home treatment team contacts, and multiple medications, showed lower proportions of remote consultation use.

**Table 2:** Use of remote consultation by year

	Cohort year						
	2019	2020	2021		2022		2023
*N*	22,226	14,895	23,655		22,864		23,235
**N of consultations mean (sd)**							
Total consultations	4.8 (6.3)	4.2 (6.2)	6.2 (7.9)		6.0 (7.4)		6.1 (7.6)
Face to face	4.8 (6.3)	3.0 (4.7)	2.8 (4.9)		3.3 (5.2)		3.8 (5.7)
Remote	0.0 (0.3)	1.1 (4.1)	3.4 (4.8)		2.6 (3.9)		2.3 (3.4)
Use of remote consultations during Q1 (%)*							
Low	98.8	66.8	26.9		34.9		38.9
Medium	1.0	10.7	25.7		28.4		30.0
High	0.2	22.5	47.4		36.7		31.1

*Levels of remote consultation use defined based on tertiles derived from Q1 data between 2020 and 2023.

On average, within the low contact category, 93.1% of patients had zero remote consultations during the Q1 period. The specific sample sizes for this non-exposed subgroup across all study years are detailed in Supplementary Table 2.

### Associations between the use of remote consultations and clinical outcomes

Logistic regression analyses displayed in Table 3 showed that higher proportions of remote consultations were associated with lower hospitalisations, emergency assessments, and home treatment team contacts, particularly in earlier cohorts. However, in later years and after adjusting for baseline severity, these associations attenuated, suggesting non-inferiority of remote care compared to in-person care, with Odds Ratios (ORs) approaching 1.00. Associations with ORs > 1.00 lost significance when applying Bonferroni-corrected *p*-values for multiple comparisons, as described in Supplementary Table 3.

**Table 3:** Logistic regression for the associations of outcome measures with 10% increments in the proportion of remote consultation use for each cohort

	Cohort year											
	2020^a^			2021^b^			2022^c^			2023 ^d^		
	OR	95% CI	p	OR	95% CI	p	OR	95% CI	p	OR	95% CI	p
**Hospitalisation**												
Unadjusted	**0.93**	**[0.91-0.95]**	**0.000**	**0.98**	**[0.97-0.99]**	**0.002**	0.99	[0.98-1.00]	0.054	**0.99**	**[0.97-1.00]**	**0.040**
Model 1	**0.93**	**[0.91-0.94]**	**0.000**	**0.97**	**[0.96-0.99]**	**0.000**	**0.98**	**[0.97-1.00]**	**0.010**	0.99	[0.97-1.00]	0.125
Model 2	**0.93**	**[0.91-0.95]**	**0.000**	**0.97**	**[0.96-0.99]**	**0.000**	**0.98**	**[0.97-1.00]**	**0.016**	0.99	[0.98-1.01]	0.240
Model 3	**0.96**	**[0.94-0.98]**	**0.000**	**0.98**	**[0.96-0.99]**	**0.009**	0.99	[0.97-1.00]	0.144	1.00	[0.98-1.02]	0.964
Model 4	1.00	[0.97-1.02]	0.756	1.02	[1.00-1.04]	0.086	1.02	[1.00-1.03]	0.089	**1.02**	**[1.01-1.04]**	**0.013**
**Emergency assessment**												
Unadjusted	**0.94**	**[0.93-0.96]**	**0.000**	**0.97**	**[0.96-0.98]**	**0.000**	**0.98**	**[0.96-0.99]**	**0.000**	**0.98**	**[0.97-0.99]**	**0.000**
Model 1	**0.92**	**[0.91-0.94]**	**0.000**	**0.95**	**[0.94-0.96]**	**0.000**	**0.96**	**[0.95-0.98]**	**0.000**	**0.97**	**[0.96-0.98]**	**0.000**
Model 2	**0.92**	**[0.90-0.94]**	**0.000**	**0.95**	**[0.94-0.96]**	**0.000**	**0.96**	**[0.95-0.97]**	**0.000**	**0.97**	**[0.96-0.98]**	**0.000**
Model 3	**0.92**	**[0.91-0.94]**	**0.000**	**0.95**	**[0.93-0.96]**	**0.000**	**0.96**	**[0.95-0.98]**	**0.000**	**0.97**	**[0.96-0.99]**	**0.000**
Model 4	**0.98**	**[0.96-1.00]**	**0.023**	1.00	[0.98-1.02]	0.931	1.01	[1.00-1.03]	0.082	**1.02**	**[1.00-1.04]**	**0.015**
**Home treatment team**												
Unadjusted	**0.93**	**[0.92-0.95]**	**0.000**	**0.98**	**[0.97-0.99]**	**0.001**	**0.98**	**[0.97-0.99]**	**0.008**	**0.99**	**[0.97-1.00]**	**0.044**
Model 1	**0.92**	**[0.91-0.94]**	**0.000**	**0.96**	**[0.95-0.98]**	**0.000**	**0.97**	**[0.96-0.98]**	**0.000**	**0.98**	**[0.96-0.99]**	**0.002**
Model 2	**0.92**	**[0.90-0.94]**	**0.000**	**0.96**	**[0.94-0.97]**	**0.000**	**0.97**	**[0.95-0.98]**	**0.000**	**0.98**	**[0.96-0.99]**	**0.002**
Model 3	**0.93**	**[0.92-0.95]**	**0.000**	**0.96**	**[0.94-0.97]**	**0.000**	**0.97**	**[0.95-0.98]**	**0.000**	**0.97**	**[0.96-0.99]**	**0.003**
Model 4	**0.98**	**[0.96-1.00]**	**0.038**	0.99	[0.98-1.01]	0.497	1.00	[0.98-1.02]	0.990	1.00	[0.99-1.02]	0.644
**Improvement**												
Unadjusted	**1.02**	**[1.01-1.03]**	**0.000**	**1.03**	**[1.02-1.04]**	**0.000**	1.00	[1.00-1.01]	0.146	1.01	[1.00-1.01]	0.100
Model 1	**1.02**	**[1.01-1.02]**	**0.001**	**1.02**	**[1.01-1.03]**	**0.000**	1.00	[0.99-1.01]	0.760	1.00	[0.99-1.01]	0.964
Model 2	**1.02**	**[1.01-1.03]**	**0.000**	**1.02**	**[1.02-1.03]**	**0.000**	1.00	[0.99-1.01]	0.624	1.00	[0.99-1.01]	0.963
Model 3	**1.03**	**[1.02-1.04]**	**0.000**	**1.02**	**[1.01-1.03]**	**0.000**	1.00	[0.99-1.00]	0.246	1.00	[0.99-1.01]	0.832
Model 4	**1.05**	**[1.04-1.06]**	**0.000**	**1.03**	**[1.02-1.04]**	**0.000**	1.00	[0.99-1.01]	0.995	1.00	[1.00-1.01]	0.446
**Deterioration**												
Unadjusted	**0.98**	**[0.98-0.99]**	**0.001**	**1.02**	**[1.01-1.02]**	**0.000**	1.00	[1.00-1.01]	0.347	**1.01**	**[1.00-1.02]**	**0.001**
Model 1	**0.99**	**[0.98-0.99]**	**0.002**	**1.02**	**[1.01-1.02]**	**0.000**	1.01	[1.00-1.01]	0.169	**1.01**	**[1.00-1.02]**	**0.003**
Model 2	**0.99**	**[0.98-1.00]**	**0.008**	**1.02**	**[1.01-1.02]**	**0.000**	1.00	[1.00-1.01]	0.224	**1.01**	**[1.01-1.02]**	**0.001**
Model 3	0.99	[0.98-1.00]	0.189	1.01	[1.00-1.01]	0.128	0.99	[0.99-1.00]	0.225	1.01	[1.00-1.01]	0.177
Model 4	**1.02**	**[1.01-1.03]**	**0.001**	**1.02**	**[1.02-1.03]**	**0.000**	1.01	[1.00-1.02]	0.013	**1.02**	**[1.01-1.03]**	**0.000**

Adjustments. Unadjusted: Crude. Model 1: Age, sex, ethnic group, IMD. Model 2: Marital status, loneliness, long-term conditions, mobility impairment, hearing impairment, visual impairment. Model 3: Diagnosis. Model 4: Emergency assessments, hospitalisations, HTT, medication.

OR Odds ratio, CI Confidence interval.

Statistically significant associations in bold (*p* < 0.05).

^a^Unadjusted *N* = 14,779; adjusted *N* = 14,304.

^b^Unadjusted *N* = 23,517; adjusted *N* = 22,910.

^c^Unadjusted *N* = 22,707; adjusted *N* = 22,042.

^d^Unadjusted *N* = 23,067; adjusted *N* = 22,285.

Results regarding mentions of improvement and deterioration were more nuanced. While a significant association was found between the proportion of remote consultation use and recorded mentions of improvement in earlier cohorts (2020–2021), remote care was also consistently associated with higher mentions of deterioration across all cohorts. These associations remained significant when applying Bonferroni-corrected *p*-values (see Supplementary Table 3). Given the exploratory nature of these natural language processing (NLP) measures, which capture broad clinical fluctuations across domains such as behaviour and social functioning, these associations are interpreted as hypothesis-generating rather than confirmatory. Outcome percentages for varying degrees of remote consultation use by year are presented in Supplementary Table 4.

Further logistic regression analyses stratifying by psychiatric diagnosis revealed significant heterogeneity across conditions, as shown in Table 4.

Fully adjusted models confirmed that there were no consistent associations between remote consultation use and adverse outcomes for the majority of diagnostic groups, including dementia (F00–F03), bipolar disorder (F30–F31), depressive disorders (F32–F39), phobias and anxiety disorders (F40–F41), and personality disorders (F60–F61).

**Table 4:** Logistic regression for the associations of outcome measures with 10% increments in the proportion of remote consultation use by diagnosis and year

	Cohort year											
Use of remote consultations (continuum)*	2020			2021			2022			2023		
	OR	95% CI	p	OR	95% CI	p	OR	95% CI	p	OR	95% CI	p
F00-F03: Dementia												
Hospitalisation	0.84	[0.58-1.23]	0.371	0.93	[0.76-1.13]	0.459	1.05	[0.88-1.24]	0.608	1.02	[0.88-1.19]	0.762
Emergency assessment	NA			1.04	[0.83-1.30]	0.748	1.10	[0.93-1.31]	0.27	1.00	[0.87-1.16]	0.982
Home treatment team	0.89	[0.71-1.12]	0.322	0.93	[0.83-1.06]	0.27	0.97	[0.86-1.09]	0.602	0.92	[0.78-1.08]	0.293
Improvement	0.98	[0.93-1.03]	0.438	1.000	[0.97-1.03]	0.923	0.98	[0.95-1.01]	0.127	1.01	[0.98-1.04]	0.581
Deterioration	1	[0.94-1.05]	0.891	0.99	[0.97-1.03]	0.735	0.99	[0.96-1.02]	0.574	0.99	[0.96-1.01]	0.313
F10-F19: Mental and behavioural disorders due to psychoactive substance use												
Hospitalisation	0.98	[0.89-1.09]	0.755	1.09	[1.00-1.18]	0.052	0.99	[0.91-1.09]	0.906	1.09	[1.00-1.19]	0.052
Emergency assessment	1.01	[0.96-1.07]	0.616	1.01	[0.96-1.06]	0.749	1.04	[0.99-1.09]	0.139	**1.12**	**[1.07-1.18]**	**0.000**
Home treatment team	1.02	[0.92-1.13]	0.675	0.98	[0.91-1.07]	0.708	1.08	[0.99-1.17]	0.072	**1.11**	**[1.01-1.23]**	**0.031**
Improvement	1.02	[0.99-1.04]	0.121	1.01	[0.99-1.03]	0.587	0.98	[0.96-1.00]	0.073	0.98	[0.96-1.01]	0.208
Deterioration	1.02	[0.99-1.05]	0.146	**1.03**	**[1.01-1.06]**	**0.014**	1.03	[0.99-1.06]	0.104	**1.05**	**[1.01-1.08]**	**0.004**
F20-F29: Schizophrenia, schizotypal and delusional disorders												
Hospitalisation	**1.06**	**[1.02-1.09]**	**0.001**	**1.06**	**[1.03-1.09]**	**0.000**	**1.04**	**[1.01-1.07]**	**0.003**	**1.06**	**[1.03-1.09]**	**0.000**
Emergency assessment	1.03	[0.99-1.07]	0.110	**1.05**	**[1.02-1.08]**	**0.001**	**1.05**	**[1.02-1.08]**	**0.001**	**1.04**	**[1.01-1.07]**	**0.009**
Home treatment team	1.03	[0.99-1.06]	0.177	1.03	[1.00-1.06]	0.064	1.01	[0.98-1.04]	0.484	1.01	[0.98-1.05]	0.412
Improvement	**1.13**	**[1.10-1.16]**	**0.000**	**1.07**	**[1.05-1.08]**	**0.000**	**1.03**	**[1.01-1.05]**	**0.001**	1.00	[0.98-1.02]	0.725
Deterioration	**1.03**	**[1.01-1.06]**	**0.007**	**1.06**	**[1.04-1.08]**	**0.000**	**1.04**	**[1.03-1.06]**	**0.000**	**1.05**	**[1.02-1.07]**	**0.000**
F30-F31: Manic episode and bipolar affective disorder												
Hospitalisation	1.01	[0.95-1.08]	0.709	0.97	[0.91-1.03]	0.261	1.02	[0.96-1.09]	0.499	1.04	[0.97-1.11]	0.264
Emergency assessment	1.01	[0.95-1.08]	0.763	0.98	[0.92-1.05]	0.639	1.00	[0.95-1.07]	0.896	1.06	[1.00-1.12]	0.065
Home treatment team	1.01	[0.95-1.08]	0.693	0.98	[0.92-1.04]	0.415	1.02	[0.96-1.08]	0.54	1.02	[0.96-1.09]	0.471
Improvement	**1.10**	**[1.05-1.16]**	**0.000**	1.03	[1.00-1.07]	0.112	1.01	[0.97-1.05]	0.676	1.03	[0.99-1.07]	0.098
Deterioration	**1.06**	**[1.01-1.11]**	**0.013**	1.01	[0.97-1.05]	0.541	**1.06**	**[1.02-1.11]**	**0.003**	1.03	[0.99-1.08]	0.159
F32-F39: Depressive episode, recurrent depressive disorder and other mood affective disorders												
Hospitalisation	0.93	[0.84-1.02]	0.131	0.94	[0.87-1.02]	0.129	1.00	[0.92-1.09]	0.988	0.97	[0.88-1.06]	0.491
Emergency assessment	0.94	[0.88-1.01]	0.111	0.99	[0.93-1.05]	0.685	1.01	[0.96-1.07]	0.676	1.03	[0.97-1.09]	0.321
Home treatment team	0.94	[0.88-1.01]	0.080	0.94	[0.89-1.00]	0.044	1.01	[0.95-1.07]	0.875	0.99	[0.93-1.05]	0.680
Improvement	1.03	[0.99-1.06]	0.152	1.01	[0.99-1.04]	0.398	1.01	[0.98-1.03]	0.539	1.00	[0.98-1.03]	0.882
Deterioration	1.00	[0.97-1.04]	0.934	0.98	[0.96-1.01]	0.205	0.98	[0.95-1.00]	0.089	1.02	[0.99-1.05]	0.289
F40-F41: Phobic and other anxiety disorders												
Hospitalisation	0.73	[0.53-1.02]	0.064	0.96	[0.84-1.09]	0.508	1.12	[0.98-1.29]	0.093	0.88	[0.77-1.01]	0.066
Emergency assessment	**0.73**	**[0.55-0.96]**	**0.027**	0.98	[0.91-1.07]	0.716	1.02	[0.93-1.11]	0.690	**0.91**	**[0.83-0.99]**	**0.029**
Home treatment team	0.89	[0.75-1.07]	0.228	**0.89**	**[0.81-0.98]**	**0.022**	1.00	[0.91-1.09]	0.994	0.97	[0.88-1.07]	0.537
Improvement	1.01	[0.97-1.06]	0.598	1.01	[0.97-1.05]	0.770	**0.95**	**[0.92-0.98]**	**0.004**	0.98	[0.95-1.01]	0.251
Deterioration	**0.95**	**[0.90-1.00]**	**0.049**	1.00	[0.96-1.04]	0.876	0.99	[0.95-1.02]	0.453	1.01	[0.98-1.05]	0.498
F60-F61: Specific, mixed and other personality disorders												
Hospitalisation	0.96	[0.89-1.03]	0.275	1.05	[0.96-1.13]	0.279	0.96	[0.89-1.05]	0.392	0.95	[0.88-1.03]	0.240
Emergency assessment	0.97	[0.92-1.02]	0.208	**0.92**	**[0.88-0.96]**	**0.000**	**0.95**	**[0.90-1.00]**	**0.041**	1.00	[0.95-1.05]	0.930
Home treatment team	1.03	[0.96-1.10]	0.386	1.000	[0.94-1.06]	0.978	0.95	[0.88-1.01]	0.111	1.00	[0.94-1.06]	0.903
Improvement	1.03	[0.99-1.07]	0.141	1.01	[0.98-1.05]	0.498	**0.96**	**[0.92-0.99]**	**0.011**	0.99	[0.95-1.02]	0.471
Deterioration	1.03	[0.99-1.07]	0.210	1.03	[0.99-1.07]	0.162	0.98	[0.95-1.02]	0.353	1.03	[0.99-1.06]	0.157
Unspecified												
Hospitalisation	0.95	[0.87-1.05]	0.314	0.95	[0.90-1.00]	0.058	0.99	[0.94-1.04]	0.686	1.00	[0.95-1.06]	0.877
Emergency assessment	0.96	[0.91-1.01]	0.139	**0.96**	**[0.93-1.00]**	**0.028**	0.98	[0.94-1.01]	0.207	0.98	[0.94-1.01]	0.203
Home treatment team	**0.89**	**[0.82-0.97]**	**0.006**	0.98	[0.93-1.02]	0.29	0.98	[0.94-1.03]	0.430	0.99	[0.94-1.03]	0.568
Improvement	**1.08**	**[1.04-1.11]**	**0.000**	**1.05**	**[1.03-1.07]**	**0.000**	1.02	[0.99-1.04]	0.137	0.99	[0.97-1.02]	0.644
Deterioration	1.01	[0.97-1.04]	0.657	**1.03**	**[1.01-1.06]**	**0.006**	1.02	[0.99-1.04]	0.204	1.02	[1.00-1.05]	0.093

*Adjustments. Model 4: Age, sex, ethnic group, IMD, marital status, loneliness, long-term conditions, mobility impairment, hearing impairment, visual impairment, emergency assessments, hospitalisations, HTT, medication.

*OR* Odds ratio, *CI* Confidence interval, *NA* Not applicable.

Statistically significant associations in bold (*p* < 0.05).

However, a distinct pattern emerged for schizophrenia-related disorders (F20-F29). Unlike other psychiatric diagnoses, higher remote consultation use in this group was significantly associated with increased odds of psychiatric hospitalisations and emergency assessments across cohort years. In the most recent cohort year, the increased odds were 1.06 for psychiatric hospitalisations (95% CI 1.03–1.09) and 1.04 (95% CI 1.01–1.07) for emergency assessments for every 10% increment in the proportion of remote consultation use. Mentions of improvement and deterioration remained contradictory for this group, as both seem to increase with higher remote consultation use. All associations remained statistically significant after applying Bonferroni correction for multiple comparisons, as detailed in Supplementary Table 5.

In the final study year, there was a significant increase in emergency assessments, home treatment team contacts and deterioration mentions associated with higher remote consultation use among patients with mental and behavioural disorders due to substance use (F10–F19), but this association was not consistently present in previous years. However, the association for home treatment contacts was no longer significant after applying Bonferroni correction.

Our additional analyses showed that, after adjusting for follow-up days, the associations remained consistent with the primary models, showing increased odds of hospitalisation (OR 1.02, 95% CI 1.00–1.04) and emergency assessment (OR 1.02, 95% CI 1.00–1.04) in 2023 (see Supplementary Table 6). There was also a consistent and significant reduction in the odds of mortality across all years for patients with higher remote care utilization, reaching an OR of 0.94 (95% CI 0.91–0.97) in the most recent cohort (see Supplementary Table 7). Finally, when stratifying by referral recency, the association between remote consultation use and increased service utilization was most pronounced among long-standing referrals, particularly for hospitalizations in 2022 (OR 1.04, 95% CI 1.01–1.07) and 2023 (OR 1.03, 95% CI 1.00–1.06), while recent referrals showed an initial protective association in 2020 that stabilised over time. Clinical trajectories showed that higher remote care use remained significantly associated with increased mentions of both improvement and deterioration across multiple cohort years (see Supplementary Table 8).

## Discussion

This study provides one of the first large-scale evaluations of a profound shift in psychiatric service delivery: a transformation comparable to the deinstitutionalisation movement. However, unlike previous reforms, this change occurred rapidly and was guided by necessity due to the COVID-19 pandemic emergency. Our findings indicate that the widespread adoption of remote consultations appears safe and non-inferior for the majority of patients with mental health conditions, with the exception of those with schizophrenia-related disorders, who faced increased odds of psychiatric hospitalisations and emergency contacts. This suggests that the ‘one-size-fits-all’ approach currently being implemented in remote care may be leaving this patient group unprotected.

Our findings suggest that remote consultations are not associated with worse clinical outcomes for most psychiatric diagnoses. While there is limited evidence measuring these specific outcomes across the diagnostic spectrum, previous studies seem to be aligned with our overall results. For example, recent meta-analyses have established that remote and face-to-face consultations show no difference in symptom reduction among patients with any psychiatric diagnosis^4,5,17^. Furthermore, a meta-analysis of 32 randomised controlled trials found that telepsychiatry was as effective as, or more effective than, face-to-face psychiatry in reducing symptoms for depressive disorders, dementia, post-traumatic stress disorder, obsessive-compulsive disorder, and substance misuse, as well as for pooled diagnoses^4^. These consistent findings support the continued use of remote mental health care as a core component of service delivery. Our additional analyses also revealed a consistent and significant reduction in mortality odds among patients with higher remote care utilisation across all study years, further supporting the safety of this modality.

Nevertheless, patients diagnosed with schizophrenia-related disorders (F20-F29) represented a clear exception. Although prior research suggests that remote consultations were linked to a decrease in hospitalisations and emergency contacts before and during the COVID-19 pandemic in Spain^15^, Canada^18^, and the United States (US)^16^, this divergence likely reflects changes in the role of remote consultations. Before the pandemic, remote care was likely used selectively for patients who were clinically stable or who faced barriers to accessing standard services. During the pandemic, remote care had to be implemented almost universally. In contrast, our study captures a period of systemic implementation where remote care became a default modality.

In this context, specific mechanisms may explain the adverse outcomes observed in this group. First, the loss of non-verbal and environmental cues, such as olfactory signs of self-neglect or visible deterioration of the home environment, may compromise the clinician’s ability to detect relapse via video or phone. Second, digital tools may exacerbate paranoid ideation or impede therapeutic alliance among individuals with psychosis, creating a diagnosis-specific barrier to effective care^19,20^. These risks may be particularly pronounced among long-standing referrals, where our subgroup analyses showed the strongest associations between remote consultation use and increased hospitalisations.

In addition, the literature on substance use disorders presents an inconclusive picture. For example, a study involving opioid users in Canada reported increased hospitalisations and emergency contacts for patients engaged in telemedicine compared to face-to-face^21^. Conversely, studies focusing on opioid users in the US^22^ and alcohol users in Denmark^23^ found no significant differences. Our findings did not clearly indicate a consistent trend for this diagnostic group across study years, though emerging risks in emergency assessments and home treatment contacts were noted in the final cohort year.

Regarding clinical trajectories, mentions of improvement and deterioration were contradictory in our analysis, often increasing simultaneously with remote use. These findings should be interpreted as hypothesis-generating rather than confirmatory, given the exploratory nature of this NLP approach. The method is subject to documentation bias, as increased remote contact likely leads to more frequent note-taking overall, potentially inflating mentions of both improvement or deterioration^24^.

Furthermore, our findings on the use of remote consultations showed a nuanced perspective on digital exclusion in mental healthcare. For example, it has been largely documented that older age groups tend to use remote care less than younger groups^25^, which is in line with our findings. However, contrary to other studies^26^, populations living in more deprived areas used remote mental health consultations more than those living in less deprived areas. This could suggest that access to remote care might mitigate certain structural barriers, such as costs associated with travel, childcare or time taken off work.

The present study offers the first comprehensive evaluation of the impact of remote consultations on individuals with various mental health diagnoses using extensive real-world data. This design captures the reality of service delivery rather than the controlled conditions of a trial. Furthermore, instead of contrasting fully in-person consultations with entirely remote consultations, our analysis considers the full range of remote consultation usage, better reflecting current practice. Unlike previous studies that focus on a singular diagnosis or aggregate all patients, this study provides a nuanced perspective by stratifying outcomes across the diagnostic spectrum.

Nonetheless, this study also has limitations. First, while our design captures service provision shifts within SLaM, it does not allow to address the influence of external service changes or access to services from other providers (e.g., due to out-migration). Second, the study cannot account for factors influencing a clinician’s decision to provide remote or face-to-face consultations, which may act as a confounder. Third, in the absence of standardised symptom severity scores, we used mentions of improvement and deterioration as proxy indicators of clinical progress; however, we acknowledge that they are subject to the consistency of clinician documentation and should be treated as exploratory. Fourth, patients who died during the exposure period were excluded from the analysis, which restricts the sample to those who survived and may introduce immortality time bias. Fifth, the study lacks data on the specific mode of remote consultation (e.g., video versus telephone), which may have distinct effects on clinical outcomes and limits the granularity of our conclusions. Sixth, these results from a large urban London trust may have limited generalisability to rural or international settings.

Patient and public involvement (PPI) shaped several aspects of this study. Their feedback directed our analysis toward diagnosis-stratified outcomes and highlighted the importance of examining differences by ethnicity and mobility. This input strengthened the clinical relevance of our findings. However, PPI engagement also has inherent limitations. Panel members may not be representative of the full range of patients seen within SLaM, particularly those with more severe or complex presentations, and feedback was sought at specific time points rather than embedded continuously throughout the research process.

Our findings provide evidence that the implementation of remote care in service delivery ought to be stratified by diagnosis. Service guidelines should offer diagnosis-specific protocols for telehealth. While remote care is a valuable and safe tool for most psychiatric disorders, caution should be taken when treating patients with schizophrenia-related disorders, and potentially for those with substance misuse, to prevent missed signals that lead to hospitalisation or emergency contacts. Particular attention may be considered for patients with long-standing referrals, who appear most vulnerable to adverse outcomes associated with remote care.

Further investigations are necessary to rigorously validate these findings, particularly using electronic health records across different health systems to test generalisability. A logical next step is to examine the underlying factors contributing to the efficacy or failure of remote consultations for specific diagnoses, considering the perspectives of both clinicians and patients. Future research should also explore patient preferences and experiences to ensure that remote care delivery is equitable and acceptable across diverse populations.

The rapid transition to remote mental health care represents a fundamental and likely permanent shift in service delivery. Our large-scale analysis offers reassurance that remote care is clinically safe and non-inferior to face-to-face care for most patients with a psychiatric diagnosis. However, we identified increased odds of worse clinical outcomes for patients with schizophrenia-related disorders associated with higher proportions of remote consultations. Clinical guidelines must use a stratified approach for this diagnostic group and long-standing referrals.

## Methods

### Study design and data source

This was a retrospective study based on cohorts assembled using data obtained from the South London and Maudsley (SLaM) Clinical Research Interactive Search (CRIS) platform. SLaM provides specialist mental health services to a catchment area of approximately 1.3 million people living in the London boroughs of Croydon, Lambeth, Lewisham and Southwark. The CRIS platform was established in 2008 to render SLaM’s electronic health records available for research in a de-identified format within a robust patient-led security and information governance framework^27,28^.

Prior to the COVID-19 pandemic, remote consultations within SLaM were conducted primarily by telephone. During and after the pandemic, Microsoft Teams was introduced as the main platform for video consultations. The recording of remote consultations in the electronic health record also evolved over this period. Initially, the system did not include a dedicated option to log video calls, meaning that early remote contacts were predominantly recorded as telephone consultations. As infrastructure developed, video consultations became more consistently distinguished in clinical records.

### Participants

**Fig. 2:**
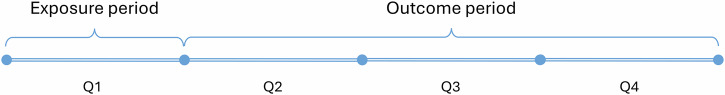
Study subperiods. Definition of exposure and outcome periods by yearly quarters (Q).

The date of the first lockdown in the United Kingdom (UK), March 26, 2020, was selected as the reference point, given the significant rise in remote consultations during the COVID-19 pandemic. Our study period spans from one year prior, starting on March 26, 2019, and concluding on March 25, 2024. Each year represented one cohort to ensure temporal relevance between the exposure and outcome periods, as well as to account for the non-linear evolution of service provision and digital infrastructure before, during and after the COVID-19 pandemic. Additionally, each year is divided into four quarters, where the first quarter (Q1) is considered the exposure period, and the subsequent three quarters (Q2, Q3, Q4) represent the outcome period (Fig. 2).

Patients were included in the cohort if they met the following inclusion criteria during Q1 of each analysed 12-month period: (a) age ≥ 18 years old at the beginning of Q1; (b) having attended at least one face-to-face or virtual clinical team contact (including outpatient and community contacts); and (c) having a recorded mental health (F-code) diagnosis, according to the International Classification of Diseases (ICD-10)^29^. Patients were excluded if they died during Q1 or if their recorded earliest accepted date at SLaM was after the end of Q1. Patients could be included in more than one year and the data were analysed separately for each cohort year.

### Outcomes, exposure and covariates

Health outcomes derived from structured fields included psychiatric hospitalisations, psychiatric assessments in emergency care, and home treatment team contacts (i.e., community-based services for patients requiring crisis support, generally daily, due to severe mental health conditions). In addition, recorded clinical trajectories were derived from mentions of improvement or deterioration in text fields of the source health records. These trajectories were identified using a rule-based natural language processing (NLP) algorithm designed to identify references from clinical text indicating that the patient is getting better or getting worse in a specific area, with a precision of 97% and recall of 76%^30^. Changes are identified across 16 areas, including general health, mental health, physical health, cognitive symptoms, behaviours, relationships, communication, and sleep, among others^30^. Each outcome measure was dichotomised based on whether there was at least one record or mention of improvement or deterioration over each 9-month outcome period.

The exposure measure was defined as a 10% increment in the proportion of remote consultations relative to the total number of consultations during Q1. Remote consultations included community/outpatient clinical contacts with mental health professionals conducted via video calls, phone calls, and text messages, ascertained from compulsory fields for clinical case notes in the source record. The total number of consultations included both remote and face-to-face community/outpatient consultations. Additionally, we calculated follow-up days by subtracting the number of days deceased from the total outcome period.

Demographic variables were obtained from the record nearest the start of each Q1, except for age, which was obtained at the beginning of Q1. These variables included age, sex, ethnic group, and Index of Multiple Deprivation (IMD) scores, a widely used Census-derived measure of neighbourhood socioeconomic status applied to lower layer super output areas (LSOAs; approximately 1500 residents). The scores are transformed into percentiles, with lower scores indicating greater deprivation^31^. Social variables included marital status, categorised as “no partner” (including single, divorced, separated, widowed), “partner” (married, civil partner, cohabiting), and “not stated”, as well as recorded loneliness occurring between Q1 and Q4, captured using a bespoke machine-learning NLP algorithm (Cohen’s K: 81%; precision: 87%; recall: 100%)^30,32^.

Clinical variables included mental and physical health conditions. We included the psychiatric diagnosis recorded closest to the beginning of Q1, classified according to the ICD-10^29^. Diagnoses were categorised as “F00-F03: Dementia”, “F04-F09: Other disorders due to substances, brain damage or physical disease”, “F10-F19: Mental and behavioural disorders due to psychoactive substance use”, “F20-F29: Schizophrenia, schizotypal and delusional disorders”, “F30-F31: Manic episode and bipolar affective disorder”, “F32-F39: Depressive episode, recurrent depressive disorder and other mood affective disorders”, “F40-F41: Phobic and other anxiety disorders“, “F43: Reaction to severe stress, and adjustment disorders“, “F50: Eating disorders“, “F60-61: Specific, mixed and other personality disorders”, “Other diagnoses”, and “Unspecified”. The recency of the referral was derived from tertiles based on the number of days since the earliest date since the patient was accepted at SLaM, and categorised as recent, intermediate, and long-standing. For physical health, we included health records of mobility, hearing, and visual impairments from structured fields, as well as the number of recorded long-term conditions identified by an NLP algorithm (Cohen’s K 91%). The machine-learning NLP tool identifies mentions of 21 physical health conditions, including arthritis, asthma, atrial fibrillation, cerebrovascular accident, chronic kidney disease, chronic liver disease, chronic obstructive pulmonary disease, chronic sinusitis, coronary arteriosclerosis, diabetes mellitus, eczema, epilepsy, heart failure, systemic arterial hypertension, inflammatory bowel disease, ischemic heart disease, migraine, multiple sclerosis, myocardial infarction, Parkinson’s disease, psoriasis, and transient ischemic attack^30^.

Finally, to assess baseline severity of the mental health condition, we retrieved data from Q1 and the preceding three months. This data included instances of at least one record of psychiatric hospitalisation, psychiatric assessment in emergency care, or home treatment team contact from structured fields. We also ascertained the recorded use of psychotropic medication antipsychotic or antidepressant medications, mood stabilisers, or sedatives, by adding up the number of classes of psychotropics, which were each identified using a rule-based NLP algorithm with precision ranging from 90% to 98%^30^. A summary of the variables, their sources and the periods from which they were extracted is provided in Supplementary Table 9.

### Statistical analysis

The analysis was performed using Stata version 18^33^. Descriptive statistics were used to characterise the sample, and chi-square tests were used to assess differences among patients with three levels of remote consultation use (low, medium, high) defined based on tertiles derived from Q1 data collected between 2020 and 2023 (excluding 2019 due to the minimal number of remote consultations conducted that year).

Complete-case logistic regression models were used to quantify odds ratios (ORs) for the association between the five outcomes (psychiatric hospitalisation, emergency assessment, home treatment team contact, improvement and deterioration) in relation to each 10% increment in the proportion of remote consultations received. Our analysis included four models. Model 1 adjusted for demographic factors (age, sex, ethnic group, IMD), Model 2 adjusted for social and clinical factors (marital status, loneliness, long-term conditions, mobility, hearing and visual impairment), Model 3 adjusted for psychiatric diagnosis, and Model 4 adjusted for mental health severity (psychiatric hospitalisations, emergency assessment, home treatment team contact, medication). We calculated 95% confidence intervals (CIs) and *p*-values for ORs, considering *p*-values < 0.05 as statistically significant. Exact *p*-values are reported throughout. Model 4 from the primary analysis was stratified by psychiatric diagnosis for diagnoses with a sample size of at least 1000 patients in at least one of the study years.

To account for multiple comparisons across the five outcomes, a Bonferroni-corrected threshold of *p* < 0.01 was applied in the sensitivity analysis. Finally, three additional analyses were conducted: one adjusting for follow-up days to account for mortality in the outcome period, another stratifying by referral recency, and the third including mortality as an outcome.

### Ethical considerations

This study has been conducted in accordance with the Declaration of Helsinki. CRIS has received ethical approval from the South Central - Oxford C Research Ethics Committee (REC) as a database for secondary research (REC reference 23/SC/0257). This study received approval by CRIS (reference number: 22-040) on 8 June 2022. Participant consent was not needed as we used a secondary research database which uses pseudonymised data. This study follows the STrengthening the Reporting of OBservational studies in Epidemiology (STROBE) guidelines (see Supplementary Table 10).

### Patient and Public Involvement

This study received feedback from the NIHR Applied Research Collaboration (ARC) South London Public Research Panel to ensure that the research aims aligned with the interests of patients and the public. Feedback was initially sought during the writing of the funding application on September 28, 2023, during the data extraction phase on July 31, 2024, and on July 30, 2025, to advise on the findings’ interpretation.

Early feedback suggested understanding which groups and conditions benefit from remote consultations, directing our research toward analysis by diagnosis. The panel was particularly interested in identifying differences across groups according to ethnicity, mobility, and patients’ preference.

## Supplementary information

Supplementary Materials

## Data Availability

The data that support the findings of this study are pseudonymised electronic health records from the South London and Maudsley NHS Foundation Trust (CRIS). Due to ethical and information governance restrictions regarding patient confidentiality, these data are not publicly available. Access to the data is restricted to researchers with appropriate honorary contracts and approvals using a secure firewall. Information regarding the CRIS system and the formal application procedures required for external researchers can be found at: https://www.maudsleybrc.nihr.ac.uk/facilities/clinical-record-interactive-search-cris/.

## Code availability

Analytic scripts used to generate the final results presented in the manuscript, including data cleaning and analysis, have been deposited in the Zenodo repository under the identifier: 10.5281/zenodo.20071924^34^.
