# Supplementary material for: Evaluating the shift in psychiatric care: Associations between remote consultation use and clinical outcomes in a large longitudinal cohort
Source: NPJ Digit Med. 2026 Jun 26;9:637. doi: 10.1038/s41746-026-02922-w (PMC13487146; doi:10.1038/s41746-026-02922-w)
Supplement: Supplementary file 1 [file 41746_2026_2922_MOESM1_ESM.pdf]

Supplementary Materials

Contents

Supplementary Table 1: Sample demographics by consultation modality use 2

Supplementary Table 2: Proportion of no exposure within low exposure 6

Supplementary Table 3: Logistic regression for the associations of outcome measures with 10% increments in the proportion of remote consultation use for each cohort (Bonferroni-corrected) 7

Supplementary Table 4: Outcome percentages by consultation modality use 10

Supplementary Table 5: Logistic regression for the associations of outcome measures with 10% increments in the proportion of remote consultation use by diagnosis (Bonferroni-corrected) 11

Supplementary Table 6: Logistic regression adjusting for follow-up days 14

Supplementary Table 7: Logistic regression for mortality 15

Supplementary Table 8: Logistic regression by time since referral 16

Supplementary Table 9: Definition of variables 17

Supplementary Table 10: STROBE Statement Checklist 18

Supplementary Table 1: Sample demographics by consultation modality use

	Use of remote consultations by year																			
	2019				2020				2021				2022				2023			
	N = 22,082				N = 14,779				N = 23,517				N = 22,707				N = 23,068			
Use of remote consultations (%)[a]	Low	Medium	High	p	Low	Medium	High	p	Low	Medium	High	p	Low	Medium	High	p	Low	Medium	High	p
**Age group**																				
18-25	99.6	0.3	0.1	0.000	56.2	12.0	31.8	0.000	19.2	20.2	60.6	0.000	26.6	26.2	47.1	0.000	32.9	28.1	39.0	0.000
26-34	99.0	0.8	0.3		58.7	11.7	29.6		20.9	21.5	57.6		30.0	26.3	43.7		33.7	29.6	36.8	
35-49	98.1	1.6	0.3		65.9	11.6	22.5		28.2	26.3	45.6		35.5	29.4	35.1		40.5	30.7	28.8	
50-69	98.6	1.1	0.3		73.4	9.6	16.9		31.2	29.6	39.2		40.8	29.7	29.5		45.0	29.9	25.1	
70+	99.8	0.2	0.0		84.0	6.3	9.7		35.4	30.5	34.1		38.7	28.9	32.4		37.3	31.2	31.5	
**Sex (N=105,877)**																				
Female	99.1	0.7	0.2	0.000	63.4	10.0	26.6	0.000	22.3	23.2	54.6	0.000	29.6	27.1	43.3	0.000	34.2	29.6	36.2	0.000
Male	98.5	1.3	0.3		69.9	11.3	18.8		31.9	28.4	39.7		40.7	29.8	29.5		44.2	30.3	25.5	
**Ethnic group**																				
White	98.2	1.4	0.4	0.000	62.6	11.2	26.2	0.000	28.5	23.9	47.7	0.000	37.1	26.7	36.1	0.000	41.1	28.5	30.3	0.000
Black	99.4	0.5	0.1		74.9	10.0	15.1		22.8	32.9	44.2		29.8	35.5	34.6		34.7	36.6	28.7	
Asian	99.0	0.9	0.1		66.5	12.4	21.1		26.8	25.1	48.1		33.2	29.8	37.0		37.8	31.7	30.5	
Mixed	99.0	1.0	0.0		57.6	15.5	26.9		21.6	24.5	53.9		27.9	28.2	44.0		33.8	29.3	36.9	
Other	99.0	0.8	0.2		61.5	11.2	27.4		24.2	24.3	51.5		33.7	26.6	39.7		37.0	25.7	37.3	
Not stated	99.8	0.1	0.1		71.5	6.5	22.0		33.0	18.0	49.0		43.2	16.2	40.6		45.9	17.4	36.7	
**IMD quintiles (n=103,298)**																				
1 - more deprived	98.6	1.2	0.2	0.060	62.3	10.4	27.3	0.000	30.1	22.0	47.9	0.000	38.6	23.8	37.6	0.000	40.0	25.9	34.1	0.000
2	98.6	1.2	0.3		64.3	12.1	23.5		24.9	25.8	49.3		34.2	28.3	37.5		39.0	29.9	31.0	
3	98.8	1.0	0.3		67.3	10.2	22.5		25.6	26.1	48.4		32.8	29.2	38.0		38.0	30.6	31.4	
4	98.9	1.0	0.1		68.7	10.0	21.3		26.2	26.8	47.0		33.8	30.1	36.1		37.4	32.2	30.4	
5 - less deprived	99.3	0.6	0.1		70.5	10.2	19.4		26.0	27.6	46.3		33.5	30.1	36.4		37.6	31.8	30.6	

**Marital status**																				
No partner	98.5	1.2	0.3	0.000	68.0	11.5	20.5	0.000	26.3	28.4	45.4	0.000	35.1	30.9	34.0	0.000	39.6	32.5	27.9	0.000
Partner	98.6	1.2	0.3		64.0	9.7	26.3		27.5	24.3	48.2		34.9	27.5	37.6		35.9	29.2	34.9	
Not stated	99.8	0.1	0.1		65.0	8.7	26.3		28.1	20.2	51.7		34.6	23.4	42.0		39.0	25.7	35.4	
**Loneliness**																				
Not recorded	98.8	1.0	0.2	0.894	67.0	10.2	22.7	0.002	27.9	24.3	47.8	0.000	36.0	26.8	37.2	0.000	39.9	28.3	31.8	0.000
Recorded	98.8	1.0	0.2		66.0	12.5	21.5		21.2	33.9	45.0		28.9	37.0	34.1		33.9	38.8	27.3	
**Long-term condition**																				
No	98.4	1.2	0.4	0.000	60.7	9.5	29.9	0.000	29.6	17.7	52.7	0.000	38.6	21.3	40.1	0.000	42.5	24.8	32.7	0.000
Yes	98.9	0.9	0.1		68.8	11.1	20.1		26.0	28.4	45.6		33.6	30.8	35.6		36.7	33.2	30.1	
**Mobility impairment**																				
No	98.9	0.9	0.2	0.000	66.8	10.7	22.6	0.610	26.8	25.6	47.6	0.000	34.7	28.3	36.9	0.000	38.8	29.9	31.2	0.009
Yes	95.0	4.0	1.0		68.8	11.1	20.1		32.0	30.5	37.5		44.7	29.5	25.8		44.7	31.6	23.7	
**Hearing impairment**																				
No	98.8	1.0	0.2	0.887	66.8	10.6	22.5	0.370	26.8	25.7	47.5	0.005	34.9	28.3	36.8	0.015	38.9	29.9	31.1	0.175
Yes	99.0	1.0	0.0		61.4	15.7	22.9		40.2	25.2	34.6		38.6	38.6	22.7		42.2	35.6	22.2	
**Visual impairment**																				
No	98.8	1.0	0.2	0.225	66.8	10.7	22.5	0.941	26.9	25.7	47.4	0.173	34.8	28.4	36.8	0.001	38.9	29.9	31.1	0.064
Yes	97.8	1.1	1.1		65.5	12.1	22.4		32.1	30.9	37.0		56.5	23.2	20.3		44.6	36.1	19.3	
**Diagnosis**																				
F00-F03: Dementia	100.0	0.0	0.0	0.000	80.3	7.6	12.1	0.000	28.6	27.9	43.5	0.000	31.2	27.9	40.9	0.000	30.5	30.0	39.5	0.000
F04-F09: Other disorders due to substances, brain damage or physical disease	100.0	0.0	0.0		88.9	2.6	8.5		47.1	22.7	30.3		46.1	21.6	32.3		44.8	21.8	33.5	
F10-F19: Mental and behavioural disorders due to psychoactive substance use	91.7	6.9	1.4		50.4	18.3	31.3		48.7	25.2	26.1		58.4	25.0	16.6		62.7	23.5	13.8	
F20-F29: Schizophrenia, schizotypal and delusional disorders	100.0	0.0	0.0		81.8	9.4	8.8		24.6	41.9	33.5		33.1	41.6	25.2		36.6	43.6	19.8	

F30-F31: Manic episode and bipolar affective disorder	100.0	0.0	0.0		69.4	12.3	18.3		18.0	31.1	50.9		21.9	38.9	39.1		26.1	41.8	32.1	
F32-F39: Depressive episode, recurrent depressive disorder and other mood affective disorders	99.9	0.0	0.0		61.0	9.2	29.8		21.4	22.2	56.4		31.0	26.3	42.7		37.0	29.0	34.0	
F40-F41: Phobic and other anxiety disorders	100.0	0.0	0.0		48.8	9.3	41.9		17.4	17.5	65.1		28.7	18.9	52.3		35.0	25.3	39.7	
F43: Reaction to severe stress and adjustment disorders	100.0	0.0	0.0		59.0	10.0	31.0		28.9	14.4	56.7		34.5	20.7	44.8		38.3	22.4	39.3	
F50: Eating disorders	100.0	0.0	0.0		30.2	15.8	54.0		5.5	13.8	80.7		12.1	16.3	71.5		26.9	18.3	54.8	
F60-F61: Specific, mixed and other personality disorders	99.9	0.0	0.1		48.3	13.2	38.5		17.3	18.0	64.7		21.3	27.9	50.8		29.9	34.0	36.1	
Other	99.9	0.1	0.0		53.5	7.8	38.6		19.6	12.4	67.9		30.3	17.1	52.5		35.3	17.9	46.8	
Unspecified	99.8	0.1	0.2		76.3	6.8	16.9		28.0	17.5	54.4		36.7	21.3	42.0		38.3	23.5	38.2	
**Referral recency**[b]																				
Recent	98.7	1.0	0.3	0.000	62.1	10.0	28.0	0.000	28.7	21.4	49.9	0.000	36.4	23.6	40.0	0.000	39.6	25.1	35.2	0.000
Intermediate	98.4	1.3	0.3		63.5	11.7	24.8		26.3	23.5	50.2		33.6	27.8	38.6		38.2	29.3	32.5	
Long-standing	99.4	0.6	0.0		74.5	10.4	15.1		25.7	32.2	42.1		34.7	33.4	31.9		39.0	35.0	26.0	
**Severity - hospitalization**																				
None	98.7	1.1	0.2	0.000	66.0	10.1	24.0	0.000	27.7	23.4	48.9	0.000	35.9	26.1	38.0	0.000	39.9	28.0	32.1	0.000
1+	99.8	0.2	0.0		74.7	16.3	9.1		17.0	53.2	29.8		23.8	53.6	22.6		27.7	53.9	18.4	
**Severity - emergency**																				
None	98.7	1.1	0.2	0.016	63.7	10.6	25.7	0.000	24.9	23.9	51.2	0.000	33.1	26.6	40.4	0.000	37.1	28.6	34.3	0.000
1+	99.3	0.6	0.1		83.4	11.3	5.3		39.5	37.1	23.4		45.8	39.0	15.2		50.0	38.0	12.1	
**Severity - home treatment**																				
None	98.7	1.1	0.2	0.000	65.9	10.2	23.9	0.000	27.9	23.2	49.0	0.000	36.2	25.7	38.1	0.000	39.9	27.7	32.3	0.000
1+	99.9	0.1	0.0		75.9	15.3	8.8		15.8	55.0	29.2		20.4	59.2	20.4		26.3	58.5	15.2	
**Severity – medication**																				
None	98.1	1.5	0.4	0.000	55.6	9.9	34.6	0.000	31.9	14.6	53.5	0.000	39.9	16.4	43.7	0.000	44.5	16.9	38.6	0.000
1	99.1	0.8	0.1		72.7	9.1	18.2		27.3	27.5	45.2		35.1	30.0	34.9		38.1	32.9	29.0	

2+	99.4	0.5	0.2	71.6	13.0	15.5	21.4	35.1	43.4	29.3	39.7	31.0	33.8	41.2	25.0

[a] Levels of remote consultation use defined based on tertiles derived from Q1 data between 2020 and 2023

[b] Diagnosis recency defined based on tertiles

Differences were calculated using chi-squared test

Supplementary Table 2: Proportion of no exposure within low exposure

	Year				
	2019	2020	2021	2022	2023
No exposure (N)	21,660	9,493	5,582	6,852	7,548
Low exposure (N)	21,814	9,875	6,327	7,928	8,982
Proportion of no exposure / low exposure	0.99	0.96	0.88	0.86	0.84

Supplementary Table 3: Logistic regression for the associations of outcome measures with 10% increments in the proportion of remote consultation use for each cohort (Bonferroni-corrected)

Use of remote consultations (continuum)*	Cohort year											
	2020[a]			2021[b]			2022[c]			2023[d]		
	OR	95% CI	p	OR	95% CI	p	OR	95% CI	p	OR	95% CI	p
**Hospitalisation**												
Unadjusted	**0.93**	**[0.91-0.95]**	**0.000**	**0.98**	**[0.97-0.99]**	**0.002**	0.99	[0.98-1.00]	0.054	0.99	[0.97-1.00]	0.040
Model 1	**0.93**	**[0.91-0.94]**	**0.000**	**0.97**	**[0.96-0.99]**	**0.000**	0.98	[0.97-1.00]	0.010	0.99	[0.97-1.00]	0.125
Model 2	**0.93**	**[0.91-0.95]**	**0.000**	**0.97**	**[0.96-0.99]**	**0.000**	0.98	[0.97-1.00]	0.016	0.99	[0.98-1.01]	0.240
Model 3	**0.96**	**[0.94-0.98]**	**0.000**	**0.98**	**[0.96-0.99]**	**0.009**	0.99	[0.97-1.00]	0.144	1.00	[0.98-1.02]	0.964
Model 4	1.00	[0.97-1.02]	0.756	1.02	[1.00-1.04]	0.086	1.02	[1.00-1.03]	0.089	1.02	[1.01-1.04]	0.013
**Emergency assessment**												
Unadjusted	**0.94**	**[0.93-0.96]**	**0.000**	**0.97**	**[0.96-0.98]**	**0.000**	**0.98**	**[0.96-0.99]**	**0.000**	**0.98**	**[0.97-0.99]**	**0.000**
Model 1	**0.92**	**[0.91-0.94]**	**0.000**	**0.95**	**[0.94-0.96]**	**0.000**	**0.96**	**[0.95-0.98]**	**0.000**	**0.97**	**[0.96-0.98]**	**0.000**
Model 2	**0.92**	**[0.90-0.94]**	**0.000**	**0.95**	**[0.94-0.96]**	**0.000**	**0.96**	**[0.95-0.97]**	**0.000**	**0.97**	**[0.96-0.98]**	**0.000**
Model 3	**0.92**	**[0.91-0.94]**	**0.000**	**0.95**	**[0.93-0.96]**	**0.000**	**0.96**	**[0.95-0.98]**	**0.000**	**0.97**	**[0.96-0.99]**	**0.000**
Model 4	0.98	[0.96-1.00]	0.023	1.00	[0.98-1.02]	0.931	1.01	[1.00-1.03]	0.082	1.02	[1.00-1.04]	0.015
**Home treatment team**												
Unadjusted	**0.93**	**[0.92-0.95]**	**0.000**	**0.98**	**[0.97-0.99]**	**0.001**	**0.98**	**[0.97-0.99]**	**0.008**	0.99	[0.97-1.00]	0.044
Model 1	**0.92**	**[0.91-0.94]**	**0.000**	**0.96**	**[0.95-0.98]**	**0.000**	**0.97**	**[0.96-0.98]**	**0.000**	**0.98**	**[0.96-0.99]**	**0.002**
Model 2	**0.92**	**[0.90-0.94]**	**0.000**	**0.96**	**[0.94-0.97]**	**0.000**	**0.97**	**[0.95-0.98]**	**0.000**	**0.98**	**[0.96-0.99]**	**0.002**
Model 3	**0.93**	**[0.92-0.95]**	**0.000**	**0.96**	**[0.94-0.97]**	**0.000**	**0.97**	**[0.95-0.98]**	**0.000**	**0.97**	**[0.96-0.99]**	**0.003**
Model 4	0.98	[0.96-1.00]	0.038	0.99	[0.98-1.01]	0.497	1.00	[0.98-1.02]	0.990	1.00	[0.99-1.02]	0.644
**Improvement**												
Unadjusted	**1.02**	**[1.01-1.03]**	**0.000**	**1.03**	**[1.02-1.04]**	**0.000**	1.00	[1.00-1.01]	0.146	1.01	[1.00-1.01]	0.100
Model 1	**1.02**	**[1.01-1.02]**	**0.001**	**1.02**	**[1.01-1.03]**	**0.000**	1.00	[0.99-1.01]	0.760	1.00	[0.99-1.01]	0.964

Model 2	**1.02**	**[1.01-1.03]**	**0.000**	**1.02**	**[1.02-1.03]**	**0.000**	1.00	[0.99-1.01]	0.624	1.00	[0.99-1.01]	0.963
Model 3	**1.03**	**[1.02-1.04]**	**0.000**	**1.02**	**[1.01-1.03]**	**0.000**	1.00	[0.99-1.00]	0.246	1.00	[0.99-1.01]	0.832
Model 4	**1.05**	**[1.04-1.06]**	**0.000**	**1.03**	**[1.02-1.04]**	**0.000**	1.00	[0.99-1.01]	0.995	1.00	[1.00-1.01]	0.446
**Deterioration**												
Unadjusted	**0.98**	**[0.98-0.99]**	**0.001**	**1.02**	**[1.01-1.02]**	**0.000**	1.00	[1.00-1.01]	0.347	**1.01**	**[1.00-1.02]**	**0.001**
Model 1	**0.99**	**[0.98-0.99]**	**0.002**	**1.02**	**[1.01-1.02]**	**0.000**	1.01	[1.00-1.01]	0.169	**1.01**	**[1.00-1.02]**	**0.003**
Model 2	**0.99**	**[0.98-1.00]**	**0.008**	**1.02**	**[1.01-1.02]**	**0.000**	1.00	[1.00-1.01]	0.224	**1.01**	**[1.01-1.02]**	**0.001**
Model 3	0.99	[0.98-1.00]	0.189	1.01	[1.00-1.01]	0.128	0.99	[0.99-1.00]	0.225	1.01	[1.00-1.01]	0.177
Model 4	**1.02**	**[1.01-1.03]**	**0.001**	**1.02**	**[1.02-1.03]**	**0.000**	1.01	[1.00-1.02]	0.013	**1.02**	**[1.01-1.03]**	**0.000**

Adjustments. Unadjusted: Crude. Model 1: Age, sex, ethnic group, IMD. Model 2: Marital status, loneliness, long-term conditions, mobility impairment, hearing impairment, visual impairment. Model 3: Diagnosis. Model 4: Emergency assessments, hospitalisations, HTT, medication.

OR = Odds ratio; CI = Confidence interval

Statistically significant associations in bold ($p<0.01$), applying Bonferroni-corrected p-values.

[a] Unadjusted N = 14,779; adjusted N =14,304

[b] Unadjusted N = 23,517; adjusted N =22,910

[c] Unadjusted N = 22,707; adjusted N =22,042

[d] Unadjusted N = 23,067; adjusted N =22,285

Supplementary Table 4: Outcome percentages by consultation modality use

	2019 N=22,082			2020 N=14,779			2021 N=23,517			2022 N=22,707			2023 N=23,068		
**Outcomes in Q2-Q4**	**Low**	**Medium**	**High**	**Low**	**Medium**	**High**	**Low**	**Medium**	**High**	**Low**	**Medium**	**High**	**Low**	**Medium**	**High**
**Hospitalisation (%)**															
Yes	7.1	1.8	0.0	9.4	11.2	4.4	4.5	12.1	5.5	5.4	12.0	5.6	5.1	11.4	4.8
**Emergency visits (%)**															
Yes	6.8	6.9	0.0	9.8	12.2	5.4	6.8	10.8	6.4	7.1	11.7	6.3	7.5	12.2	6.4
**Home treatment (%)**															
Yes	5.6	0.5	0.0	8.6	9.4	4.4	3.7	10.7	4.5	4.2	10.3	4.3	4.3	9.2	4.0
**Improvement (%)**															
Yes	46.6	39.4	24.0	51.1	65.0	54.5	37.2	60.4	50.0	40.0	59.7	44.8	41.1	60.1	44.3
**Deterioration (%)**															
Yes	25.3	13.8	14.0	30.0	36.5	26.7	21.3	36.0	28.3	22.0	36.6	25.0	22.5	35.5	26.0

Supplementary Table 5: Logistic regression for the associations of outcome measures with 10% increments in the proportion of remote consultation use by diagnosis (Bonferroni-corrected)

Use of remote consultations (continuum)*	Cohort year											
	2020			2021			2022			2023		
	**OR**	**95% CI**	**p**	**OR**	**95% CI**	**p**	**OR**	**95% CI**	**p**	**OR**	**95% CI**	**p**
F00-F03: Dementia												
Hospitalisation	0.84	[0.58-1.23]	0.371	0.93	[0.76-1.13]	0.459	1.05	[0.88-1.24]	0.608	1.02	[0.88-1.19]	0.762
Emergency assessment	NA			1.04	[0.83-1.30]	0.748	1.10	[0.93-1.31]	0.27	1.00	[0.87-1.16]	0.982
Home treatment team	0.89	[0.71-1.12]	0.322	0.93	[0.83-1.06]	0.27	0.97	[0.86-1.09]	0.602	0.92	[0.78-1.08]	0.293
Improvement	0.98	[0.93-1.03]	0.438	1.000	[0.97-1.03]	0.923	0.98	[0.95-1.01]	0.127	1.01	[0.98-1.04]	0.581
Deterioration	1.00	[0.94-1.05]	0.891	0.99	[0.97-1.03]	0.735	0.99	[0.96-1.02]	0.574	0.99	[0.96-1.01]	0.313
F10-F19: Mental and behavioural disorders due to psychoactive substance use												
Hospitalisation	0.98	[0.89-1.09]	0.755	1.09	[1.00-1.18]	0.052	0.99	[0.91-1.09]	0.906	1.09	[1.00-1.19]	0.052
Emergency assessment	1.01	[0.96-1.07]	0.616	1.01	[0.96-1.06]	0.749	1.04	[0.99-1.09]	0.139	**1.12**	**[1.07-1.18]**	**0.000**
Home treatment team	1.02	[0.92-1.13]	0.675	0.98	[0.91-1.07]	0.708	1.08	[0.99-1.17]	0.072	1.11	[1.01-1.23]	0.031
Improvement	1.02	[0.99-1.04]	0.121	1.01	[0.99-1.03]	0.587	0.98	[0.96-1.00]	0.073	0.98	[0.96-1.01]	0.208
Deterioration	1.02	[0.99-1.05]	0.146	1.03	[1.01-1.06]	0.014	1.03	[0.99-1.06]	0.104	**1.05**	**[1.01-1.08]**	**0.004**
F20-F29: Schizophrenia, schizotypal and delusional disorders												
Hospitalisation	**1.06**	**[1.02-1.09]**	**0.001**	**1.06**	**[1.03-1.09]**	**0.000**	**1.04**	**[1.01-1.07]**	**0.003**	**1.06**	**[1.03-1.09]**	**0.000**
Emergency assessment	1.03	[0.99-1.07]	0.110	**1.05**	**[1.02-1.08]**	**0.001**	**1.05**	**[1.02-1.08]**	**0.001**	**1.04**	**[1.01-1.07]**	**0.009**
Home treatment team	1.03	[0.99-1.06]	0.177	1.03	[1.00-1.06]	0.064	1.01	[0.98-1.04]	0.484	1.01	[0.98-1.05]	0.412
Improvement	**1.13**	**[1.10-1.16]**	**0.000**	**1.07**	**[1.05-1.08]**	**0.000**	**1.03**	**[1.01-1.05]**	**0.001**	1.00	[0.98-1.02]	0.725
Deterioration	**1.03**	**[1.01-1.06]**	**0.007**	**1.06**	**[1.04-1.08]**	**0.000**	**1.04**	**[1.03-1.06]**	**0.000**	**1.05**	**[1.02-1.07]**	**0.000**
F30-F31: Manic episode and bipolar affective disorder												
Hospitalisation	1.01	[0.95-1.08]	0.709	0.97	[0.91-1.03]	0.261	1.02	[0.96-1.09]	0.499	1.04	[0.97-1.11]	0.264
Emergency assessment	1.01	[0.95-1.08]	0.763	0.98	[0.92-1.05]	0.639	1.00	[0.95-1.07]	0.896	1.06	[1.00-1.12]	0.065
Home treatment team	1.01	[0.95-1.08]	0.693	0.98	[0.92-1.04]	0.415	1.02	[0.96-1.08]	0.54	1.02	[0.96-1.09]	0.471
Improvement	**1.10**	**[1.05-1.16]**	**0.000**	1.03	[1.00-1.07]	0.112	1.01	[0.97-1.05]	0.676	1.03	[0.99-1.07]	0.098

Deterioration	**1.06**	**[1.01-1.11]**	**0.013**	1.01	[0.97-1.05]	0.541	**1.06**	**[1.02-1.11]**	**0.003**	1.03	[0.99-1.08]	0.159
F32-F39: Depressive episode, recurrent depressive disorder and other mood affective disorders												
Hospitalisation	0.93	[0.84-1.02]	0.131	0.94	[0.87-1.02]	0.129	1.00	[0.92-1.09]	0.988	0.97	[0.88-1.06]	0.491
Emergency assessment	0.94	[0.88-1.01]	0.111	0.99	[0.93-1.05]	0.685	1.01	[0.96-1.07]	0.676	1.03	[0.97-1.09]	0.321
Home treatment team	0.94	[0.88-1.01]	0.080	0.94	[0.89-1.00]	0.044	1.01	[0.95-1.07]	0.875	0.99	[0.93-1.05]	0.680
Improvement	1.03	[0.99-1.06]	0.152	1.01	[0.99-1.04]	0.398	1.01	[0.98-1.03]	0.539	1.00	[0.98-1.03]	0.882
Deterioration	1.00	[0.97-1.04]	0.934	0.98	[0.96-1.01]	0.205	0.98	[0.95-1.00]	0.089	1.02	[0.99-1.05]	0.289
F40-F41: Phobic and other anxiety disorders												
Hospitalisation	0.73	[0.53-1.02]	0.064	0.96	[0.84-1.09]	0.508	1.12	[0.98-1.29]	0.093	0.88	[0.77-1.01]	0.066
Emergency assessment	**0.73**	**[0.55-0.96]**	**0.027**	0.98	[0.91-1.07]	0.716	1.02	[0.93-1.11]	0.690	**0.91**	**[0.83-0.99]**	**0.029**
Home treatment team	0.89	[0.75-1.07]	0.228	**0.89**	**[0.81-0.98]**	**0.022**	1.00	[0.91-1.09]	0.994	0.97	[0.88-1.07]	0.537
Improvement	1.01	[0.97-1.06]	0.598	1.01	[0.97-1.05]	0.770	**0.95**	**[0.92-0.98]**	**0.004**	0.98	[0.95-1.01]	0.251
Deterioration	**0.95**	**[0.90-1.00]**	**0.049**	1.00	[0.96-1.04]	0.876	0.99	[0.95-1.02]	0.453	1.01	[0.98-1.05]	0.498
F60-F61: Specific, mixed and other personality disorders												
Hospitalisation	0.96	[0.89-1.03]	0.275	1.05	[0.96-1.13]	0.279	0.96	[0.89-1.05]	0.392	0.95	[0.88-1.03]	0.240
Emergency assessment	0.97	[0.92-1.02]	0.208	**0.92**	**[0.88-0.96]**	**0.000**	**0.95**	**[0.90-1.00]**	**0.041**	1.00	[0.95-1.05]	0.930
Home treatment team	1.03	[0.96-1.10]	0.386	1.000	[0.94-1.06]	0.978	0.95	[0.88-1.01]	0.111	1.00	[0.94-1.06]	0.903
Improvement	1.03	[0.99-1.07]	0.141	1.01	[0.98-1.05]	0.498	**0.96**	**[0.92-0.99]**	**0.011**	0.99	[0.95-1.02]	0.471
Deterioration	1.03	[0.99-1.07]	0.210	1.03	[0.99-1.07]	0.162	0.98	[0.95-1.02]	0.353	1.03	[0.99-1.06]	0.157
Unspecified												
Hospitalisation	0.95	[0.87-1.05]	0.314	0.95	[0.90-1.00]	0.058	0.99	[0.94-1.04]	0.686	1.00	[0.95-1.06]	0.877
Emergency assessment	0.96	[0.91-1.01]	0.139	**0.96**	**[0.93-1.00]**	**0.028**	0.98	[0.94-1.01]	0.207	0.98	[0.94-1.01]	0.203
Home treatment team	**0.89**	**[0.82-0.97]**	**0.006**	0.98	[0.93-1.02]	0.29	0.98	[0.94-1.03]	0.430	0.99	[0.94-1.03]	0.568
Improvement	**1.08**	**[1.04-1.11]**	**0.000**	**1.05**	**[1.03-1.07]**	**0.000**	1.02	[0.99-1.04]	0.137	0.99	[0.97-1.02]	0.644
Deterioration	1.01	[0.97-1.04]	0.657	**1.03**	**[1.01-1.06]**	**0.006**	1.02	[0.99-1.04]	0.204	1.02	[1.00-1.05]	0.093

* Adjustments. Model 4: Age, sex, ethnic group, IMD, marital status, loneliness, long-term conditions, mobility impairment, hearing impairment, visual impairment, emergency assessments, hospitalisations, HTT, medication.

OR = Odds ratio; CI = Confidence interval; NA = Not applicable

Statistically significant associations in bold ($p<0.05$)

Supplementary Table 6: Logistic regression adjusting for follow-up days

Use of remote consultations (continuum)*	Cohort year 2020 N=14,304			2021 N=22,910			2022 N=22,042			2023 N=22,285		
	**OR**	**95% CI**	**p**	**OR**	**95% CI**	**p**	**OR**	**95% CI**	**p**	**OR**	**95% CI**	**p**
Hospitalisation	1.00	[0.97-1.02]	0.755	1.02	[1.00-1.04]	0.081	1.02	[1.00-1.03]	0.096	**1.02**	**[1.00-1.04]**	**0.016**
Emergency assessment	**0.98**	**[0.96-1.00]**	**0.023**	1.00	[0.98-1.01]	0.915	1.01	[1.00-1.03]	0.083	**1.02**	**[1.00-1.04]**	**0.017**
Home treatment team	**0.98**	**[0.96-1.00]**	**0.038**	0.99	[0.98-1.01]	0.489	1.00	[0.98-1.02]	0.999	1.00	[0.99-1.02]	0.660
Improvement	**1.05**	**[1.04-1.06]**	**0.000**	**1.03**	**[1.02-1.04]**	**0.000**	1.00	[0.99-1.01]	0.965	1.00	[1.00-1.01]	0.470
Deterioration	**1.02**	**[1.01-1.03]**	**0.001**	**1.03**	**[1.02-1.03]**	**0.000**	**1.01**	**[1.00-1.02]**	**0.012**	**1.02**	**[1.01-1.03]**	**0.000**

Adjustments. Age, sex, ethnic group, IMD, marital status, loneliness, long-term conditions, mobility impairment, hearing impairment, visual impairment, diagnosis, severity, follow-up days

OR = Odds ratio; CI = Confidence interval

Statistically significant associations in bold (p<0.05)

Supplementary Table 7: Logistic regression for mortality

**Use of remote consultations (continuum)***	**Cohort year**											
	**2020 N=14,045**			**2021 N=22,910**			**2022 N=22,042**			**2023 N=21,661**		
	**OR**	**95% CI**	**p**	**OR**	**95% CI**	**p**	**OR**	**95% CI**	**p**	**OR**	**95% CI**	**p**
Mortality in Q2-Q4	**0.95**	**[0.91-0.99]**	**0.014**	**0.97**	**[0.94-0.99]**	**0.006**	**0.96**	**[0.93-0.98]**	**0.001**	**0.94**	**[0.91-0.97]**	**0.000**

Adjustments. Age, sex, ethnic group, IMD, marital status, loneliness, long-term conditions, mobility impairment, hearing impairment, visual impairment, diagnosis, severity

OR = Odds ratio; CI = Confidence interval

Statistically significant associations in bold (p<0.05)

Supplementary Table 8: Logistic regression by time since referral

Use of remote consultations (continuum)*	Cohort year											
	2020			2021			2022			2023		
	OR	95% CI	p	OR	95% CI	p	OR	95% CI	p	OR	95% CI	p
Recent referral												
Hospitalisation	**0.93**	**[0.88-0.97]**	**0.001**	0.99	[0.96-1.03]	0.620	0.97	[0.94-1.01]	0.103	1.00	[0.96-1.04]	0.875
Emergency assessment	**0.96**	**[0.92-0.99]**	**0.017**	0.99	[0.96-1.02]	0.417	1.00	[0.97-1.03]	0.992	1.02	[0.99-1.06]	0.189
Home treatment team	**0.92**	**[0.88-0.96]**	**0.000**	0.98	[0.95-1.01]	0.167	0.97	[0.94-1.01]	0.115	1.01	[0.97-1.05]	0.706
Improvement	**1.03**	**[1.01-1.04]**	**0.001**	**1.04**	**[1.02-1.05]**	**0.000**	1.00	[0.98-1.01]	0.636	1.00	[0.99-1.01]	0.799
Deterioration	1.01	[0.99-1.03]	0.384	**1.02**	**[1.00-1.03]**	**0.014**	0.99	[0.98-1.00]	0.199	**1.02**	**[1.01-1.04]**	**0.003**
Intermediate referral												
Hospitalisation	**0.96**	**[0.93-1.00]**	**0.033**	1.01	[0.98-1.04]	0.438	1.00	[0.97-1.03]	0.794	1.02	[0.98-1.05]	0.308
Emergency assessment	**0.96**	**[0.93-0.99]**	**0.014**	0.99	[0.96-1.01]	0.238	1.00	[0.97-1.03]	0.990	1.00	[0.98-1.03]	0.937
Home treatment team	0.98	[0.94-1.01]	0.167	1.01	[0.98-1.04]	0.745	1.00	[0.97-1.04]	0.783	1.01	[0.98-1.04]	0.668
Improvement	**1.05**	**[1.03-1.07]**	**0.000**	**1.02**	**[1.01-1.04]**	**0.001**	0.99	[0.98-1.01]	0.312	1.00	[0.99-1.01]	0.946
Deterioration	1.01	[0.99-1.02]	0.476	**1.04**	**[1.02-1.05]**	**0.000**	**1.02**	**[1.01-1.03]**	**0.007**	**1.02**	**[1.01-1.04]**	**0.002**
Long-standing referral												
Hospitalisation	1.01	[0.98-1.05]	0.421	1.01	[0.98-1.03]	0.679	**1.04**	**[1.01-1.07]**	**0.003**	**1.03**	**[1.00-1.06]**	**0.026**
Emergency assessment	1.02	[0.98-1.05]	0.315	1.01	[0.98-1.03]	0.535	1.02	[0.99-1.04]	0.201	1.02	[1.00-1.05]	0.063
Home treatment team	0.99	[0.96-1.03]	0.736	0.99	[0.96-1.02]	0.366	1.01	[0.98-1.04]	0.537	1.00	[0.97-1.03]	0.975
Improvement	**1.06**	**[1.04-1.08]**	**0.000**	**1.03**	**[1.01-1.04]**	**0.000**	1.01	[1.00-1.02]	0.188	1.01	[1.00-1.02]	0.102
Deterioration	**1.04**	**[1.02-1.06]**	**0.000**	**1.05**	**[1.04-1.07]**	**0.000**	**1.05**	**[1.03-1.06]**	**0.000**	**1.03**	**[1.02-1.05]**	**0.000**

* Adjustments. Model 4: Age, sex, ethnic group, IMD, marital status, loneliness, long-term conditions, mobility impairment, hearing impairment, visual impairment, emergency assessments, hospitalisations, HTT, medication.

OR = Odds ratio; CI = Confidence interval; NA = Not applicable

Statistically significant associations in bold (p<0.05)

Supplementary Table 9: Definition of variables

Variable category	Variable name	Data source	Extraction period
**Exposure**	Remote consultation proportion	Structured fields	Q1
**Outcomes**	Psychiatric hospitalisation	Structured fields	Q2 to Q4
	Emergency psychiatric assessment	Structured fields	Q2 to Q4
	Home Treatment Team contacts	Structured fields	Q2 to Q4
	Clinical trajectories (Improvement/Deterioration)	NLP-derived from clinical text	Q2 to Q4
	Follow-up days	Derived from structured fields	Q2 to Q4
**Demographics**	Age	Structured fields	Start of Q1
	Sex	Structured fields	Closest to start of Q1
	Ethnic group	Structured fields	Closest to start of Q1
	Index of Multiple Deprivation (IMD)	Structured fields	Closest to start of Q1
**Social Factors**	Marital tatus	Structured fields	Closest to start of Q1
	Loneliness	NLP-derived from clinical text	Q1 to Q4
**Clinical Factors**	Psychiatric diagnosis	Structured fields	Closest to start of Q1
	Recency of referral	Derived from structured fields	Earliest accepted date
	Physical impairments (mobility, hearing, visual)	Structured fields	Closest to start of Q1
	Long-term conditions	NLP-derived from clinical text	Closest to start of Q1
**Baseline Severity**	Prior psychiatric service use	Structured fields	Q1 and preceding 3 months
	Psychotropic medication use	NLP-derived from clinical text	Q1 and preceding 3 months

Supplementary Table 10: STROBE Statement Checklist

Checklist of items that should be included in reports of ***cohort studies***

	Item No	Recommendation	Page No
**Title and abstract**	1	(*a*) Indicate the study's design with a commonly used term in the title or the abstract	1
		(*b*) Provide in the abstract an informative and balanced summary of what was done and what was found	2
**Introduction**			
Background/rationale	2	Explain the scientific background and rationale for the investigation being reported	2-3
Objectives	3	State specific objectives, including any prespecified hypotheses	3
**Methods**			
Study design	4	Present key elements of study design early in the paper	9
Setting	5	Describe the setting, locations, and relevant dates, including periods of recruitment, exposure, follow-up, and data collection	10
Participants	6	(*a*) Give the eligibility criteria, and the sources and methods of selection of participants. Describe methods of follow-up	10
		(*b*) For matched studies, give matching criteria and number of exposed and unexposed	N/A
Variables	7	Clearly define all outcomes, exposures, predictors, potential confounders, and effect modifiers. Give diagnostic criteria, if applicable	10-12
Data sources/ measurement	8*	For each variable of interest, give sources of data and details of methods of assessment (measurement). Describe comparability of assessment methods if there is more than one group	10-12, ST9
Bias	9	Describe any efforts to address potential sources of bias	12-13
Study size	10	Explain how the study size was arrived at	10
Quantitative variables	11	Explain how quantitative variables were handled in the analyses. If applicable, describe which groupings were chosen and why	10-13
Statistical methods	12	(*a*) Describe all statistical methods, including those used to control for confounding	12-13
		(*b*) Describe any methods used to examine subgroups and interactions	12-13
		(*c*) Explain how missing data were addressed	12
		(*d*) If applicable, explain how loss to follow-up was addressed	N/A
		(*e*) Describe any sensitivity analyses	13
**Results**			
Participants	13*	(a) Report numbers of individuals at each stage of study—eg numbers potentially eligible, examined for eligibility, confirmed eligible, included in the study, completing follow-up, and analysed	Fig 1

		(b) Give reasons for non-participation at each stage	N/A
		(c) Consider use of a flow diagram	Fig 1
Descriptive data	14*	(a) Give characteristics of study participants (eg demographic, clinical, social) and information on exposures and potential confounders	3-4
		(b) Indicate number of participants with missing data for each variable of interest	Table 1
		(c) Summarise follow-up time (eg, average and total amount)	3
Outcome data	15*	Report numbers of outcome events or summary measures over time	ST4
Main results	16	(*a*) Give unadjusted estimates and, if applicable, confounder-adjusted estimates and their precision (eg, 95% confidence interval). Make clear which confounders were adjusted for and why they were included	Table 2
		(*b*) Report category boundaries when continuous variables were categorized	Table 1
		(*c*) If relevant, consider translating estimates of relative risk into absolute risk for a meaningful time period	N/A
Other analyses	17	Report other analyses done—eg analyses of subgroups and interactions, and sensitivity analyses	ST3, ST5, ST6, ST7, ST8
**Discussion**			
Key results	18	Summarise key results with reference to study objectives	6
Limitations	19	Discuss limitations of the study, taking into account sources of potential bias or imprecision. Discuss both direction and magnitude of any potential bias	8
Interpretation	20	Give a cautious overall interpretation of results considering objectives, limitations, multiplicity of analyses, results from similar studies, and other relevant evidence	6-9
Generalisability	21	Discuss the generalisability (external validity) of the study results	9
**Other information**			
Funding	22	Give the source of funding and the role of the funders for the present study and, if applicable, for the original study on which the present article is based	14

*Give information separately for exposed and unexposed groups.

Note: An Explanation and Elaboration article discusses each checklist item and gives methodological background and published examples of transparent reporting. The STROBE checklist is best used in conjunction with this article (freely available on the Web sites of PLoS Medicine at http://www.plosmedicine.org/, Annals of Internal Medicine at http://www.annals.org/, and Epidemiology at http://www.epidem.com/). Information on the STROBE Initiative is available at http:// www.strobe-statement.org.
